# Unveiling the Potential of a New β‐Cyclodextrin‐Suxibuzone Conjugate in Proteasome Regulation

**DOI:** 10.1002/cmdc.202500401

**Published:** 2025-10-16

**Authors:** Noemi Bognanni, Stefania Zimbone, Maria Laura Giuffrida, Giuseppe Di Natale, Danilo Milardi, Graziella Vecchio, Valeria Lanza

**Affiliations:** ^1^ Dipartimento di Scienze Chimiche Università degli studi di Catania V.le A.Doria, 6 95125 Catania Italy; ^2^ Istituto di Cristallografia—Consiglio Nazionale delle Ricerche Sede Secondaria di Catania Via Paolo. Gaifami 18 95126 Catania Italy

**Keywords:** activator, cyclodextrin, glycoconjugate, proteasome, pyrazolone

## Abstract

The proteasome is a central component of the cellular machinery responsible for degrading misfolded or damaged proteins, thereby maintaining protein homeostasis. Dysregulation of proteasome activity has been implicated in various diseases, including neurodegenerative disorders and cancer. In this article, a new β‐cyclodextrin conjugate of suxibuzone (SB‐CD) is designed and its proteasome activity on purified human 20S core particle and in differentiated human neuroblastoma SH‐SY5Y cells (dSHSY5Y) is investigated. This conjugate enhances the proteolytic activity of the 20S proteasome in a dose‐dependent manner, with an increase observed at concentrations as low as 5 µM. The EC50 values for SB‐CD are determined to be 0.6 ± 0.1 µM for chymotrypsin‐like activity and 1.1 ± 0.3 µM for trypsin‐like activity, indicating higher efficacy compared to suxibuzone alone. In dSH‐SY5Y cells, a decrease in the accumulation of ubiquitinated proteins is observed, consistent with the activation of the proteasome. High‐resolution electrospray ionization mass spectrometry investigations confirmed the internalization of SB‐CD in cells and verified the stability of the conjugate in response to cellular protease effects, after incubation for up to 24 h. These promising results suggest that the new conjugate is an effective enhancer of proteasome activity, holding significant promise for therapeutic applications targeting proteasome‐related pathologies.

## Graphical abstract

A novel β‐cyclodextrin‐suxibuzone conjugate enhances chymotryptic‐like activity of the 20S proteasome in a dose‐dependent manner. It remains stable and is internalized in SH‐SY5Y cells for 24 h. These findings suggest therapeutic potential for proteasome‐related disorders through targeted proteasome activation.

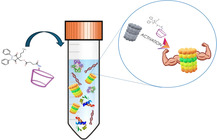

## 1 Introduction

The proteasome is a multisubunit enzyme that plays a critical role in maintaining protein quality control, and its function has been extensively studied over the past few decades, particularly concerning age‐associated declines.^[^ ^1^ ^,^ ^2^ ^]^ Inefficient proteasomes can lead to a widespread accumulation of misfolded proteins and a general decline in regulated protein turnover.^[^ ^3^ ^]^ The regulation of protein turnover is a key factor in many pathological conditions, especially proteinopathies.^[^ ^4^ ^]^ Proteinopathies refer to diseases and conditions caused by disrupted proteostasis, resulting in proteotoxic stress and altered cellular signaling and metabolism.^[^ ^4^, ^5^ ^–^ ^6^ ^]^ Numerous studies have focused on genetic and pharmacological manipulation of proteasomal activities in various animal models.^[^ ^7^, ^8^ ^–^ ^9^ ^]^ Proteasome activation may help clear abnormal protein aggregates that contribute to neuronal damage.^[^ ^6^ ^,^ ^10^ ^,^ ^11^ ^]^ Enhancements in chymotrypsin‐like and trypsin‐like proteasome activities have been observed in different cellular models.^[^ ^12^, ^13^, ^14^ ^–^ ^15^ ^]^

Various classes of compounds have been tested as proteasome activators.^[^ ^13^ ^,^ ^16^ ^]^ Dihydroquinazolines enhanced 20S proteasome activity and promoted the degradation of α‐synuclein, a protein associated with the onset and progression of Parkinson's disease.^[^ ^17^ ^]^ Natural compounds like oleuropein, derived from olive leaves and olive oil, have also demonstrated proteasome activation properties.^[^ ^18^ ^]^ These findings highlight the potential of both synthetic and natural compounds in modulating proteasome activity for therapeutic purposes.^[^ ^13^ ^,^ ^16^ ^]^

Recent studies have indicated that pyrazolones, a family of synthetic compounds, can activate the proteasome, showing potential therapeutic benefits in neurodegenerative diseases.^[^ ^19^ ^]^ A pyrazolone, TCH‐165, is a small molecule modulator of proteasome assembly, which increases 20S levels and facilitates 20S‐mediated protein degradation.^[^ ^20^ ^,^ ^21^ ^]^ Proteasome activator reversibility affects efficacy and safety, with two types: direct and indirect. Direct activators bind reversibly to the proteasome, altering its shape to enhance activity. For example, TCH‐165 opens the proteasome gate and breaks down disordered proteins without damaging the structure. Indirect activators work through signaling pathways, such as the activation of NRF1/2 or the unfolded protein response. Small molecules like TCH‐165, aminopyrine, and nifenazone share similar reversible, noncovalent binding mechanisms, enabling flexible activity regulation.^[^ ^22^ ^,^ ^23^ ^]^ Suxibuzone (SB), a prodrug of phenylbutazone, belongs to the class of pyrazolones. It is a widely used nonsteroidal anti‐inflammatory drug known for its potent analgesic and anti‐inflammatory effects.^[^ ^24^ ^]^ Because it is a drug, it offers the advantage of repurposing, bypassing the typical challenges of drug development, and significantly reducing both the time and cost associated with drug commercialization. Despite its efficacy, clinical use of SB is often hampered by poor solubility, limited bioavailability, and adverse gastrointestinal effects.^[^ ^24^, ^25^, ^26^, ^27^ ^–^ ^28^ ^]^ Modifying the SB structure could overcome these limitations. Numerous studies have shown that the conjugation of drugs with biomolecules, such as cyclodextrins, is a good strategy to improve pharmacological properties and reduce toxicity.^[^ ^29^, ^30^ ^–^ ^31^ ^]^

Cyclodextrins (CD) are cyclic oligosaccharides composed of 1,4‐linked glucopyranose units.^[^ ^32^ ^,^ ^33^ ^]^ They possess a cavity and a hydrophilic outer surface, enabling them to form inclusion complexes with a wide range of guest molecules.^[^ ^34^ ^,^ ^35^ ^]^ This unique property of CDs enhances poorly water‐soluble drug solubility, stability, and bioavailability. In addition to CD inclusion complexes, covalent conjugation has emerged as a promising approach to improve the pharmacokinetic and therapeutic profiles of several insoluble drugs.^[^ ^36^, ^37^, ^38^, ^39^, ^40^ ^–^ ^41^ ^]^ Covalent conjugates form stable chemical bonds between drugs and CD derivatives, improving solubility, controlled release, and targeted delivery. These conjugates can reduce the dosage frequency and minimize the side effects of the drug.^[^ ^34^ ^,^ ^35^ ^,^ ^42^ ^]^

**Figure 1:**
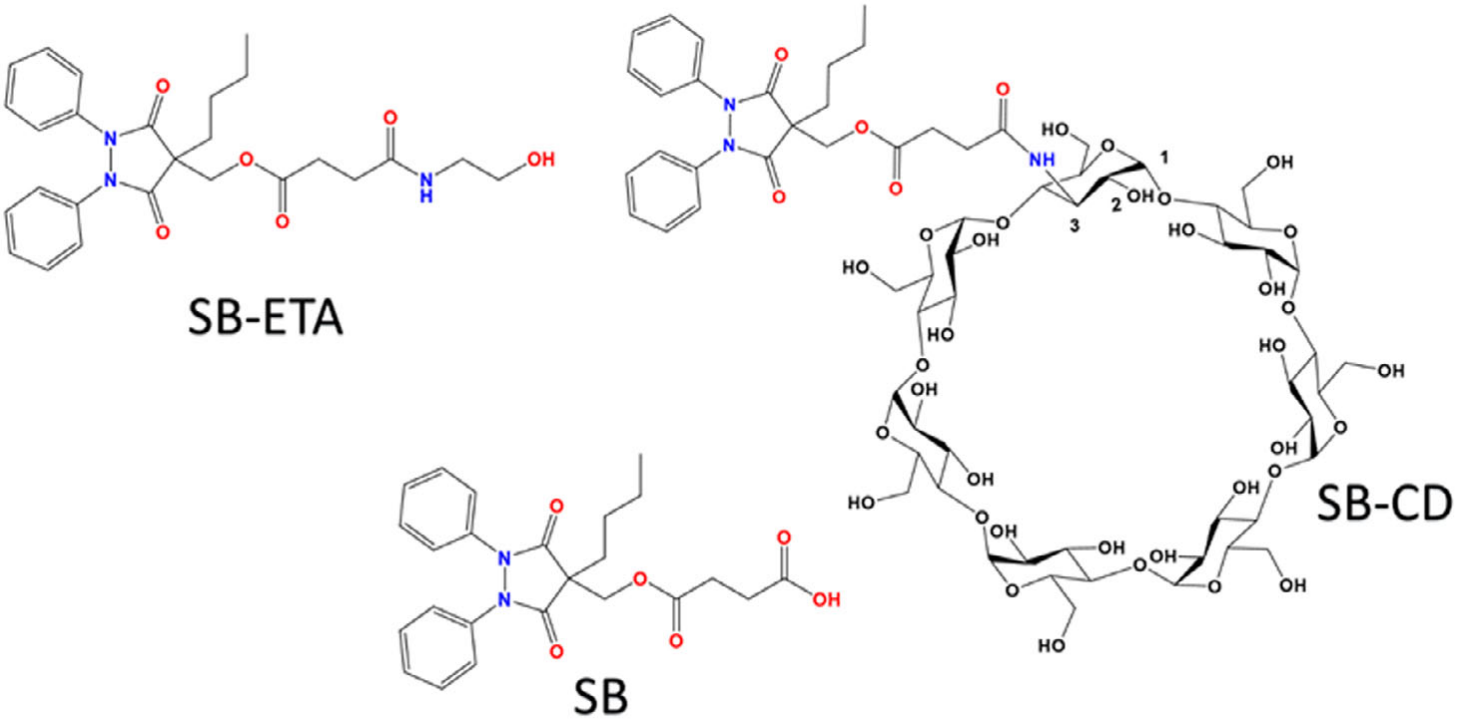
Suxibuzone (SB), amide derivative of suxibuzone (SB‐ETA), and β‐cyclodextrin derivative SB‐CD.

In this work, we designed and synthesized a novel CD conjugate of suxibuzone (SB‐CD, **Figure** **1**). The synthesis involved the conjugation of SB with 3A‐Amino‐3A‐deoxy‐(2AS, 3AS)‐β‐cyclodextrin (βCD3NH_2_) through an amide linkage. The new covalent conjugate was characterized by high‐resolution mass spectrometry (HR MS) and nuclear magnetic resonance (NMR)spectroscopy. We also synthesized an amide derivative of SB (SB‐ETA, Figure 1) for a better comparison with the CD derivative and to exclude the possibility that its behavior is due to the absence of the COOH group. Our findings from the assessment of the proteasome activity using purified 20S core and lysates from the differentiated neuroblastoma cell line, SH‐SY5Y, demonstrated that SB‐CD significantly enhanced the chymotrypsin‐like and trypsin‐like proteolytic activity of the proteasome. The internalization of the novel conjugate has been studied by mass spectrometry, confirming the presence of the studied compounds in cell lysates. This indicates the potential of SB‐CD as an effective proteasome modulator, paving the way for promising future therapeutic applications.

## 2 Results and Discussion

### 2.1 Synthesis and Characterization

The new conjugate SB‐CD was synthesized starting from βCD3NH_2_ and SB in the presence of EDC and HOBt. This functionalization can strengthen binding affinity in aqueous environments for specific guest molecules. βCD3NH2 contains an altrose unit due to the preparation methods. The synthesis scheme is reported in Figure S1, Supporting Information. The experimental high‐resolution mass spectrum (HR MS) is shown in Figure S2, Supporting Information.

**Figure 2:**
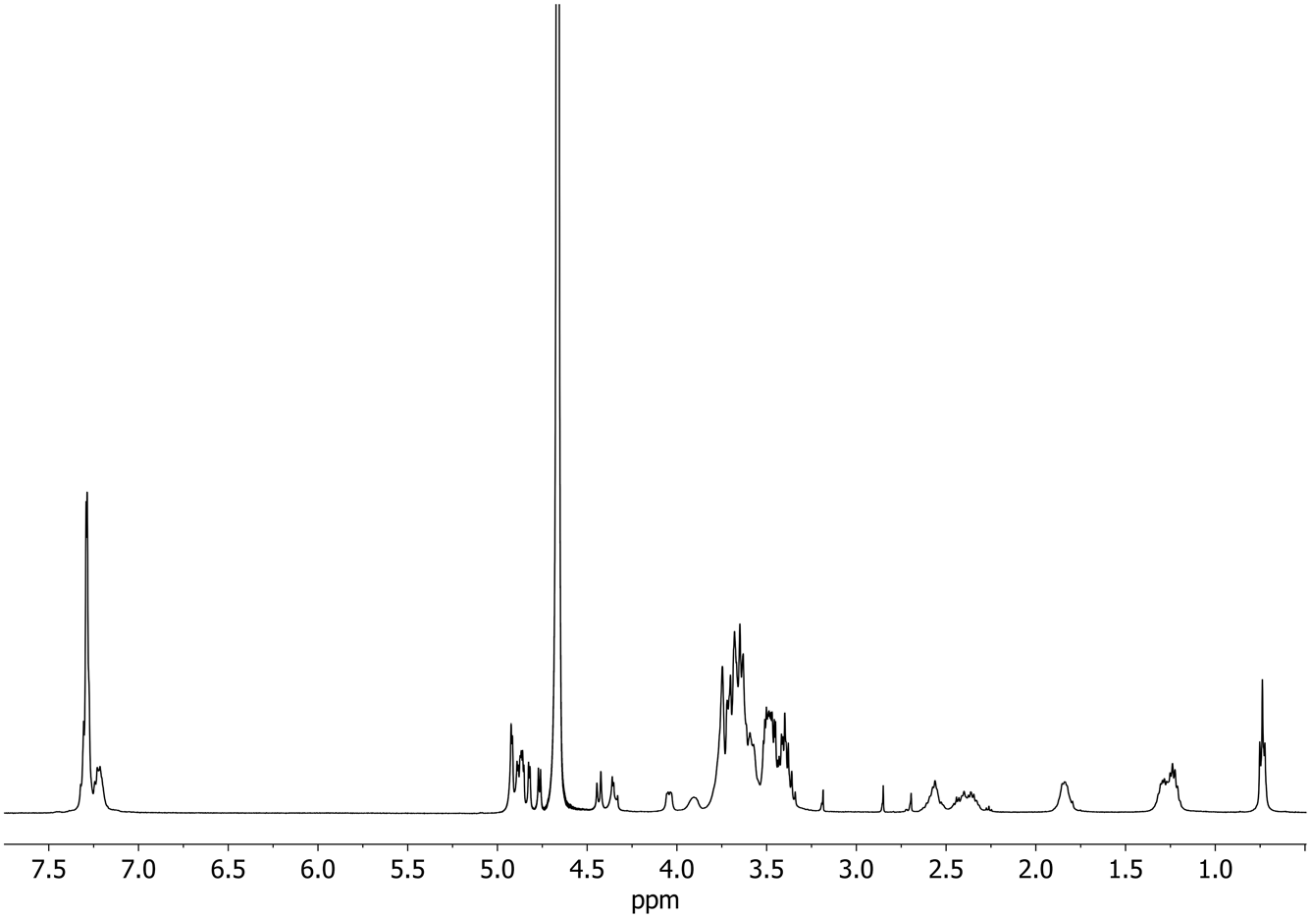
^1^H NMR spectrum (500 MHz in D_2_O) of SB‐CD (at 2.85 and 2.70 ppm DMF peaks).

The final product was characterized by NMR (**Figure** **2**, S3, S4, S5, Supporting Information). In the spectra, signals of the SB moiety are present in the aromatic region due to the phenyl rings and in the aliphatic region due to the butylene and succinyl chains. The signals of the succinic chain protons are at 2.38 and 2.57 ppm, and the diastereotopic C*H*_2_O are at 4.4 and 4.35 ppm. In the H‐1 region of CD, the signals due to the functionalized A ring can be identified at 4.77 ppm. From the 2D spectra (Figure S3, S4, S5, Supporting Information), the protons of the functionalized rings can be identified, confirming the functionalization of CD with SB.

Rotating frame overhauser effect spectroscopy (ROESY) spectra were also recorded (Figure S5, Supporting Information). Cross‐peak correlation between the aromatic protons of the SB moiety and the H‐3 and H‐5 of the CD can be observed in the spectra (Figure S5, Supporting Information), consistent with the interaction of the benzene rings with the CD cavity.

SB‐ETA was also synthesized with a condensation reaction and characterized by NMR spectra and HR MS (Figure S6, S7, Supporting Information). The presence of the ethanolamine (ETA) chain can be found in the spectra in addition to the signals of the SB moiety (Figure S6, Supporting Information).

### 2.2 Proteasome Activity on Purified Human 20S Core Particle

Among the three primary catalytic functions of the proteasome, chymotrypsin (Ch‐L), trypsin (T‐L), and caspase‐like (C‐L), Ch‐L is the most prevalent and comprises most of the proteasome's degradation capacity^[^ ^4^ ^,^ ^43^ ^,^ ^44^ ^]^ and is critical in breaking down long‐lived and regulatory proteins, directly influencing essential processes like proliferation, apoptosis, and the cellular stress response.^[^ ^45^ ^,^ ^46^ ^]^

**Figure 3:**
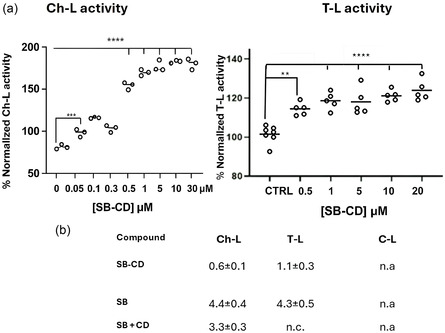
a) Activation of ChT‐L activity (left) and T‐L activity (right) of the h20S proteasome by different solutions of SB‐CD, in the concentration range 0.05–50 µM; b) EC_50_ values for chymotrypsin and trypsin activity for SB‐CD conjugate, SB alone, and a mixture of SB and βCD. (n.c. noncalculated; n.a. no activity). Graphs represent the mean ± standard error of the mean (SEM) of three independent experiments with *n* = 3. ****p < 0.001, ***p < 0.01 versus control.

The three proteolytic activities of h20S (1.5 nM) on the 7‐amino‐4‐methylcoumarin (AMC) synthetic substrates were measured in the absence and presence of conjugate over a concentration range between 50 nM and 50 µM (**Figure** **3**). The compound SB‐CD shows a selective modulatory effect on proteasomal function in relation to trypsin and chymotrypsin activity. The results highlighted that SB‐CD induced a significant (about 200%) stimulation of the cleavage kinetics of the fluorogenic substrate by 20S for Ch‐L activity (Figure 3a, left). Analysis of trypsin‐like activity reveals a clear dose‐dependent increase, with activity levels rising up to ≈120%–125% compared to control values as the concentration increases from 0.5 to 20 µM (Figure 3a, right). Conversely, caspase‐like activity remains essentially unchanged under the same conditions. The selective activation of the chymotryptic and trypsin‐like activity, while leaving the caspase‐like site unaffected, indicates that SB‐CD may act through an allosteric mechanism that preferentially alters conformational states governing basic residue cleavage (Figure S9a,b, Supporting Information). This selective modulation is particularly relevant because the balance between different proteasome activities affects overall protein homeostasis. Enhancing trypsin‐like and potentially chymotrypsin‐like activities could facilitate the clearance of misfolded or aggregation‐prone proteins while minimizing unwanted effects on regulatory proteolysis.^[^ ^13^ ^]^

The EC50 values were calculated using a dose–response nonlinear fit, (Equation 1), as described in Experimental Section. The EC50 values (Figure 3) for SB‐CD were 0.6 ± 0.1 µM for Ch‐L activity and 1.1 ± 0.3 µM for T‐L activity. These values were significantly lower than the value for SB alone, which was 4.4 ± 0.4 µM and 4.3 ± 0.5 µM for Ch‐L and T‐L activities, respectively (Figure 3b, Figure S8 a,b Supporting Information).

To rule out the possibility that the enhanced activation effect was caused by the absence of the free COOH group, the activities of SB‐ETA, Ch‐L, T‐L, and C‐L at 5 and 10 μM were compared to the same concentrations of SB alone. The SB‐ETA was less soluble than suxibuzone, which limited the concentrations that could be tested. Our results showed that both test compounds had activity similar to suxibuzone, with no statistically significant difference (Figure S9c, Supporting Information).

It has been confirmed that free cyclodextrin, at the same concentration range, did not affect proteasome activity (Figure S10, Supporting Information). The corresponding mixture of SB + CD (ratio 1:1) was used as a comparison in all experiments and EC_50_ value was calculated for Ch‐L activity (Figure 3b). Covalent bonding with CD enhances the compound's effectiveness, suggesting a more favorable interaction with the biological target or improved stability/concentration of the active drug compared to the simple physical mixture (Figure S8c, Supporting Information).

### 2.3 Stimulation of Proteasome Activity on SH‐SY5Y Cell Lysates

The promising results observed with purified human 20S proteasome led to an investigation of whether activation remains detectable in cell lysates in the presence of all possible physiological proteasome recognition motifs. To rule out any cytotoxic effect of the tested compounds, 3‐(4,5‐dimethylthiazol‐2‐yl)‐2,5‐diphenyltetrazolium bromide (MTT) experiments on the fully differentiated SH‐SY5Y cell line were carried out (Figure S7, Supporting Information). This neuronal‐like model was also used to analyze the effect of the conjugate on the 20S activity in cell lysates. Specifically, SB‐CD was administered at 37 °C to lysates of untreated SH‐SY5Y cells at the concentration range previously reported. Then, the amount of fluorogenic substrates was added, and the fluorescence intensity at 460 nm was monitored for 1 h.

**Figure 4:**
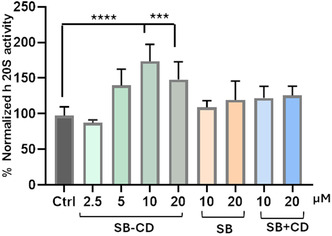
Normalized chymotryptic activities versus control on SH‐SY5Y lysates. The SB‐CD, SB, and a mixture of SB and CD were preincubated in two different concentration ranges: 2.5–20 µM for conjugate, 10–20 µM for SB, and SB + CD for 15 min, and then the fluorogenic substrate was added. Bars represent the mean ± SEM of three independent experiments with *n* = 3. ****p < 0.001, ***p < 0.01 versus control.

The data show that the SB‐CD significantly enhances proteasome activity in a dose‐dependent manner, with the highest activity observed at 10 µM. In contrast, SB also increases activity, although to a lesser extent than the conjugate (**Figure** **4**). The statistical significance indicated by asterisks suggests that the observed differences are highly significant, particularly at higher concentrations of SB‐CD.

The increase in proteasome activity observed in the lysate of SH‐SY5Y cells is particularly noteworthy. Indeed, this result indicates that SB‐CD can effectively enhance proteasome function directly within a cellular environment. This is crucial because it suggests that SB‐CD could modulate proteasome activity in living cells, a key factor for its therapeutic application.

### 2.4 Effect on the Accumulation of Ubiquitinated Proteins in Cell Culture

The increase in proteasome activity, as revealed by the fluorogenic substrate in the lysate of SH‐SY5Y cells, has also been explored through the evaluation of the level of ubiquitinated proteins following treatment. Accumulation of ubiquitinated proteins indicates impaired degradation or increased demand on the proteasome, whereas a decrease suggests active proteasomal degradation.

To test whether the SB‐CD compound was able to induce a decrease in poly‐ubiquitinated proteins, their levels were assessed in dSH‐SY5Y lysates following 6 h of treatment with the concentration (10 µM) of the conjugate, which exhibited the highest chymotryptic activity in the in vitro assay.

**Figure 5:**
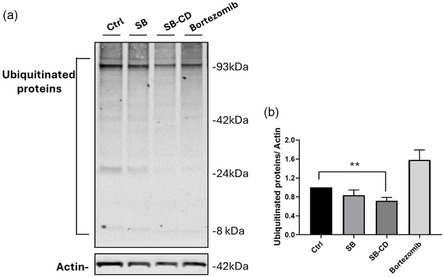
Western blot analysis of ubiquitinated proteins. a) Representative Western blot of differentiated SH‐SY5Y (dSH‐SY5Y) lysates in basal condition (Ctrl) or following 6 h treatment with 10 μM Suxibuzone (SB), and β‐Cyclodextrin‐Suxibuzone (SB‐CD). Bortezomib has been used as a control of Proteasome blocking. b) Histogram shows densitometric analysis of total ubiquitinated proteins, normalized on actin levels. Bars represent means ± SEM of four independent experiments with *n* = 3. **p < 0.05 SB‐CD versus Ctrl by unpaired T‐Test.

Western blot analysis revealed that SB‐CD led to a decrease in anti‐ubiquitin antibody signal compared to control (**Figure** **5**a,b). Notably, this reduction was more pronounced than that observed with the unmodified Suxibuzone. In contrast, as expected, treatment with Bortezomib resulted in an increase in ubiquitinated proteins, consistent with proteasome inhibition.

### 2.5 Liquid Chromatography Mass Spectrometry (LC‐MS) Investigations

To assess SB‐CD's capacity to cross the plasma membrane, LC and HR electrospray ionization mass spectrometry (HR‐ESI‐MS) were used. Samples for LC‐MS analysis were collected from lysates of fully differentiated SH‐SY5Y cells treated with SB‐CD or SB (10 µM). Lysates from untreated cells were also examined as a negative control. The LC‐MS analyses were carried out using a linear gradient tailored to ensure good separation between analytes detected by the targeted selected ion monitoring (SIM) acquisition method (see Experimental Section). Target ions were individually and sequentially isolated in predefined *m*/*z* ranges and retention time (RT) windows by a quadrupole analyzer while mass spectra were acquired by an Orbitrap mass analyzer.

**Figure 6:**
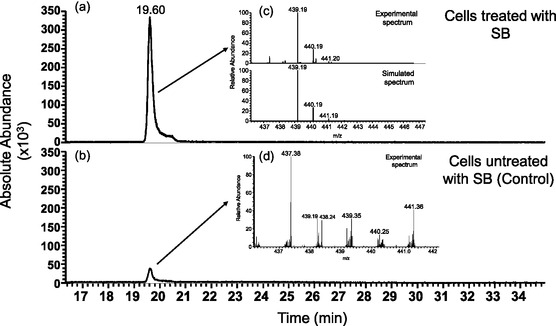
Chromatographic profiles reported as XIC (*m*/*z* = 439.18–439.20) of a) lysates obtained from SH‐SY5Y cells treated with SB and b) lysates obtained from SH‐SY5Y cells not treated with SB. The inset c) compares the experimental and theoretical isotopic distribution of the *m*/*z* peak corresponding to the SB. Simulated spectrum was calculated for SB molecular formula: C_24_H_26_O_6_N_2._ The inset d) shows the mass spectrum corresponding to the chromatographic peak observed at RT = 19.6 min of a control sample.

**Figure 7:**
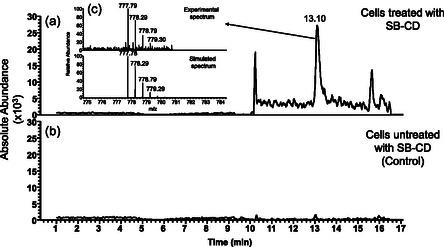
Chromatographic profiles reported as XIC (*m*/*z* = 777.75–777.8) of a) lysates obtained from SH‐SY5Y cells treated with SB‐CD and b) lysates obtained from SH‐SY5Y cells not treated with SB‐CD. The inset c) compares the experimental and theoretical isotopic distribution of the *m*/*z* peak corresponding to the SB‐CD. The simulated spectrum was calculated for the SB‐CD molecular formula: C_66_H_95_O_39_N_3_.

The extracted‐ion chromatograms (XIC) reported in **Figure** **6** indicate the presence of SB in the cell lysates treated with SB. The analysis of the samples obtained from untreated cells (controls) revealed the presence of a chromatographic peak at the same RT (19.6 min). Nevertheless, the mass spectrum corresponding to the peak observed showed only a trace of SB coming from residual amounts of standard compound injected into the column during chromatographic optimization. Detection of the SB signal even after several successive void runs could be ascribed both to the hydrophobicity of the molecule and the high sensitivity of the acquisition method (targeted SIM) applied in mass spectrometry analysis. Interestingly, LC‐MS analysis of samples obtained from differentiated SH‐SY5Y cells treated with SB‐CD revealed a signal corresponding to the SB conjugate (**Figure** **7**), suggesting the capability of SB‐CD to cross the membrane.

**Figure 8:**
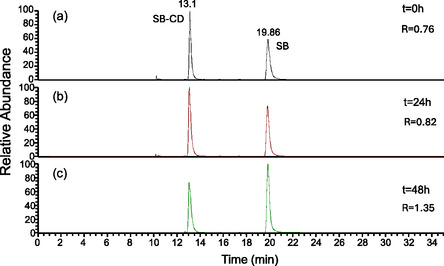
Chromatographic profiles reported as XIC (*m*/*z* = 439.18–439.20 and *m*/*z* = 777.75–777.8) of SB and SB‐CD compounds (CSB = CSB‐CD = 1 µM) added to SH‐SY5Y cell lysates. Chromatographic profiles were acquired at different incubation times, namely a) t = 0, b) t = 24h, c) t = 48.  The ratio between the peak areas (R) of SB and SB‐CD was reported for each profile.

LC‐MS measurements were performed on cell lysates obtained from differentiated SH‐SY5Y exposed to SB or SB‐CD compounds to evaluate the stability of SB‐CD molecules. No protease inhibitors were used to preserve the enzymatic proteolytic activity. Samples were incubated at 37 °C and analyzed by LC‐MS at different incubation times. Peak areas, corresponding to SB (R = 20 min) and SB‐CD (R = 13 min) molecules, calculated by XIC recorded at *t* = 0 h, *t* = 24 h, and *t* = 48 h (**Figure** **8**), were considered to assess changes in standard concentrations caused by the degradation of the SB‐CD and SB in the lysate environment. To overcome the low reproducibility of *m*/*z* signal intensities of mass spectra acquired at different incubation times, the ratio of peak areas (SB/SB‐CD) was considered.

The increase in the calculated ratio between the areas of the two peaks corresponding to SB and SB‐CD, respectively (SB/SB‐CD), as the incubation time elapsed, could be attributed to the only partial hydrolysis of the amide bond linking SB to cyclodextrin and the resulting formation of SB. However, SB‐CD is very stable to proteolytic enzymes, and after 48 h, it is present in the medium.

## 3 Conclusion

In this study, we synthesized and characterized the new cyclodextrin conjugate of suxibuzone (SB‐CD), a drug belonging to the pyrazolone family. Biological assays demonstrated that the new conjugate significantly enhances the chymotrypsin‐like and trypsin‐like activities of the human 20S proteasome, exhibiting EC50 values (0.6 ± 0.1 µM for Ch‐L activity, 1.1 ± 0.3 µM for T‐L activity) that are markedly lower than those of suxibuzone alone. This suggests that covalent conjugation with cyclodextrin improved the bioactivity of suxibuzone. The conjugate can cross the cell membrane and remains stable in the cytosol for many hours. The high efficacy of the conjugate in modulating proteasome activity may be due to its interaction with the proteasome, suggesting that the compound could bind to the alpha3‐alpha4 grooves of the RP‐binding surface, similar to the other studied pyrazolones. Studies on differentiated SH‐SY5Y cell lysates confirmed that SB‐CD significantly stimulates proteasome activity in a dose‐dependent manner, indicating its potential efficacy in modulating proteasome function within a cellular environment. SB‐CD treatment accelerated the cell's protein degradation process by enhancing the activity or efficiency of the 20S proteasome, as observed in dSHSY5Y cell culture. Furthermore, suxibuzone‐cyclodextrin conjugate is not toxic in differentiated SH‐SY5Y cells, reinforcing its potential for use in biological systems as a proteasome modulator.

## 4 Experimental Section

4.1

4.1.1

βCD3NH_2_ was purchased from TCI Chemicals; N‐ethyl‐N′‐(3‐dimethylaminopropyl)carbodiimide hydrochloride (EDC), 1‐hydroxybenzotriazole (HOBt), ethanolamine (ETA), and tris(hydroxymethyl) aminomethane (Tris) from Fluka; and SB from Santa Cruz Biotechnology. Isolated and purified human proteasome (h20S) was purchased from Boston Biochem. Thin‐layer chromatography (TLC) was carried out in 0.20 mm‐thick silica gel 60‐layer plates with UV254 fluorescent indicator (Alugram Xtra Sil G UV254, manufacturer Macherey‐Nagel).

##### NMR Spectroscopy

^1^H and ^13^C NMR spectra were recorded at 25 °C with a Varian UNITY PLUS‐500 spectrometer at 499.9 MHz and at 125 MHz, using standard pulse programs from the Varian library. 2D experiments, correlation spectroscopy (COSY), and total correlation spectroscopy (TOCSY) were performed using 1K data points and 256 increments.

##### LC and HRMS

An UltiMate 3000 Proteomics LC instrument, equipped with a Q‐Exactive Orbitrap mass spectrometer (Thermo Fisher Scientific instrument) and controlled by the Chromeleon and Xcalibur (Thermo Scientific software), was used for sample analyses. Samples were analyzed by gradient elution with A (0.01% formic acid in water) and B (0.01% formic acid in 80% acetonitrile /20% water) with an EASY‐Spray PepSwift Monolithic (25 cm length, 200 μm internal diameter) at a microflow rate of 1.5 µL min^−^^1^. SB and SB‐CD standard solutions were used to optimize the linear gradient and improve chromatographic separation of analytes. Samples were injected into the column equilibrated to 70% A, then a linear gradient from 30% to 50% B over 15 min was performed. Finally, an isocratic gradient at 70% A was applied over 10 min to restore the initial elution conditions. The separation was monitored by ESI‐MS. The instrumental parameters for spectra acquired in the positive‐ion mode were as follows: spray voltage = 2.0 kV, capillary temperature = 200 °C; S‐lens RF level = 60 V, Sheath gas = 7; and resolving power = 140,000 FWHM. The analytes of interest were detected by targeted SIM acquisition method. Target ions were individually and sequentially isolated in predefined *m*/*z* ranges and RT windows (SB‐CD; *m*/*z* range: 774.78–780.78 (double charge); 1–16.5 min), (SB; *m*/*z* range: 436.18–442.18 (single charge); 16.35–35 min) by a quadrupole analyzer whereas mass spectra were acquired by Orbitrap mass analyzer. The chromatographic profiles recorded as raw files were analyzed using Qual Browser (Thermo Scientific) software.

##### Synthesis of SB‐CD

βCD3NH_2_ (100 mg, 8.8 × 10^−5^ mol) was added to SB (39 mg, 8.8 × 10^−5^ mol) previously activated with HOBt and EDC (16 and 21 mg, 1.1 × 10^−4^ mol) in DMF, and the solution was stirred at 25 °C. After 24 h, the solvent was evaporated, and the solid was purified by flash chromatography using an Rp18 column and a linear gradient of water‐methanol as the eluent.

Yield: 50%. TLC: Rf = 0.83 PrOH/AcEt/H_2_O/NH_3_ 5:2:2:1.

^1^H NMR: (500 MHz, D_2_O) (*δ*, ppm) 0.74 (m, 3H, CH_3_ del SB); 1.35–1.15 (m, 4H, CH_2_ del SB); 1.83 (m, 2H, CH_2_ SB), 2.38 (m, 2H, CH_2_ succinic linker del SB); 2.57 (m, 2H, CH_2_ succinic linker del SB), 3.20–3.54 (m, 14 H, H‐2, ‐4 CD); 3.54–3.84 (m, 28 H, H‐5, −6, −3 CD); 3.91 (m, 1 H, H‐5A CD); 4.02 (m, 1H, H‐3A CD); 4.44 (d, 1H, J_a, b_ = 4.44 Hz, CHaO SB); 4.35 (d, 1H, J_b, a_ = 4.43 Hz, CH_b_O SB); 4.76 (d, 1H, J_1A, 2A_ = 5.91 Hz, H‐1A CD); 4.82 (d, 1H, J_1X,2X_ = 3.99 Hz), 4.84– 4.79 (6H, H‐1 CD), 7.4–7.1 (m, 10 H, SB).

^13^C NMR: (125 MHz, D_2_O) (*δ*, ppm): 13.34 (CH_3_), 21.81 (CH_2_), 25.61 (CH_2_), 30.16 (CH_2_ succinic linker), 30.55 (CH_2_ succinic linker), 31.04 (CH_2_), 54.40 (C SB), 60.14 (C‐6 CD), 60.48 (CH_2_O SB), 73.96–71.00 (C‐5, C‐3 CD), 80.90–81.34 (C‐2 and C‐4 CD), 102–104 (C‐1 CD), 125.39 (CH C‐3‐phenyl), 128.85 (CH C‐4 phenyl SB), 129.88 (CH C‐2 phenyl SB), 132.73 (C‐1 phenyl SB), 171.03 (CO succinic ester SB), 173.97 (CON SB), 183.12 (CON succinic amide),

HR ESI MS [obsd: ([SB‐CD + 2H]^2+^ = 777.791 m/z calculated for mass for C_66_H_95_O_39_N_3_ = 777.784 m/z.

##### Synthesis of SB‐ETA

ETA (17 mg, 2.7 × 10^−^^4^ mol) was added to SB (40 mg, 9.1 × 10^−^^5^ mol) previously activated with HOBt and EDC (37 and 52 mg, 2.7  × 10^−^^4^ mol) in DMF and the solution was stirred at 25 °C. After 24 h, the solvent was evaporated, and the solid was purified by flash chromatography using a silica column eluted with a linear gradient of AcOEt/CH_3_OH.

TLC: Rf = 0.87 PrOH/AcEt/MetOH 4:3:3

^1^H NMR: (500 MHz, MeOD) (*δ*, ppm): 0.89 (m, 3H, CH_3_ SB); 1.22–1.42 (m, 4H, CH_2_ SB), 1.80–1.92 (m, 2H, CH_2_ SB), 2.41 (t, 2H, J = 7.04 Hz, CH_2_ succinic linker SB); 2.56 (t, 2H, J = 7.02 Hz, CH_2_ succinic linker SB), 3.26 (t, J = 5.56 Hz, 2H, CH_2_OH ETA), 3.56 (t, J = 5.58 Hz, CH_2_NH ETA), 4.40 (s, 2H, CH_2_O SB), 7.22 (t, 2H, J = 7.54 Hz H‐4 phenyl SB), 7.35 (t, 4H, J = 8.03 Hz, H‐2 phenyl SB), 7.38 (t, 4H, J = 8.04 Hz, phenyl SB).

^13^C NMR: (125 MHz, MeOD) (*δ*, ppm): 12.62 (CH_3_), 22,27 (CH_2_), 26.26 (CH_2_), 28.81 (CH_2_ succinic linker), 29.81 (CH_2_ succinic linker), 30.71 CH_2_), 41.45 (CH_2_NH SB), 54.07 (C SB), 60.09 (CH_2_OH), 66.00 (CH_2_O SB), 123.39 (CH C‐3‐phenyl), 127.38 (CH C‐4 phenyl SB), 128.64 (CH C‐2 phenyl SB), 135.18 (C‐1 phenyl SB), 171.46 (CON SB), 171.88 (CO succinic ester SB), 172.23 (CO succinic amide SB).

##### Determination of Proteolytic Activity on Purified Human 20S Proteasome

Proteasome activity on human 20S proteasome was determined using a microplate fluorimetric assay, as previously described.^[^ ^34^ ^]^ All assays were performed by preincubating the tested compounds (concentration range: 0.05–50 μM) in 50 mM Tris‐HCl (pH 8) at 37 °C for 15 min; then, h20S proteasome was added at a final concentration of 1.5 nM and incubated at 37 °C for 1 h. Next, 50 μM of fluorogenic peptides Suc‐LLVY‐AMC, Z‐LLG‐AMC, or Ac‐RLR‐AMC, specific for chymotrypsin‐like (ChT‐L), trypsin‐like (T‐L), and C ‐L peptidase activities respectively, were added to the proteasome solutions. The release of aminomethylcoumarin (AMC) was monitored continuously for 45 min by measuring fluorescence at 440 nm (excitation at 360 nm) by Victor Nivo, in a 384‐well plate. The EC_50_ values were calculated using the following equation that describes a dose–response nonlinear fit of data in function of activator concentration

(1) $$\%R=\frac{R\text{max}}{\left(1+{\left(EC50/X\right)}^{\mathrm{Hillslope}}\right)}$$

where *R*max is the maximum value of system response, %*R* is a percentage of maximum response, and *x* is the concentration of activator.

##### Cytotoxicity Assays

SB‐CD activity was tested on the differentiated neuroblastoma cell line, SH‐SY5Y. Cells were maintained in DMEM‐F12 (Gibco, ThermoFisher) supplemented with 10% heat‐inactivated (HI) fetal calf serum (Gibco, ThermoFisher), 100 mg mL^−1^ penicillin, and streptomycin (Gibco, ThermoFisher), and 2 mM L‐glutamine at 37 °C, 5% CO_2_. Two weeks before experiments, cells were harvested, and 5 × 10^3^ cells were plated on 96‐well plates in DMEM‐F12 with 5% HI fetal calf serum. The percentage of serum was gradually decreased until it was 1% of the total. Cells were exposed to 5 μM of all‐trans‐retinoic acid (RA) to promote neuronal differentiation. The medium‐containing RA was changed every 3 days. Fully differentiated SH‐SY5Y cells were then treated with increasing concentrations of SB‐CD (0.5, 2, 5, 10, 30, and 50 µM), and their activity was compared to that of the parent compounds (SB and SB + CD) at the same concentrations. Untreated cells were also included in the experiment.

After 48 h of treatment, cell cultures were incubated with MTT (5 mg mL^−1^ stock solution) for 3 h at 37 °C and then lysed with DMSO; the formazan production was evaluated by the Multimode Microplate Reader ‘Victor Nivo’ (Revvity Inc), through the absorbance at 570 nm.

##### Preparation of Cell Lysates

SH‐SY5Y cells were grown in DMEM F12 supplemented with Fetal Calf Serum 10% and maintained at 37 °C in a humidified 5% CO_2_ incubator. Two weeks before the experiments, cells were differentiated according to the protocol described in the previous section. Whole‐cell lysates of untreated RA‐differentiated cells were prepared by freeze−thawing cycles in water containing 1 mM dithiothreitol (DTT). The lysates were centrifuged at 10,000 rpm for 20 min, and the supernatant was brought to 50 mM Tris‐HCl (pH 7.4).

The BCA protein Assay Kit (Pierce‐Thermofisher) was used to quantify proteins, and an equal amount of proteins (4 µg) was used to test proteasomal activity in cell lysates.

##### Determination of Proteasomal Activity in Cell Lysates

The 20S proteasomal activity in lysates was determined using the same fluorogenic substrate. In every well, an amount of cell lysate (4 µg total protein) was added in Tris‐HCl 50 mM and incubated with different concentrations of SB‐CD and SB. The chymotryptic activity was measured after 15 min of incubation, adding the same fluorogenic substrate used with the purified 20S core particle, and monitored for 1 h. MG132 (4 µM), a known inhibitor of h‐20S, was used as a control.

##### Western Blot Analysis of Ubiquitinated Proteins

30 µg of total proteins from whole cell lysates of dSHSY5Y in basal condition (ctrl) or treated for 6 h with 10 μM Suxibuzone (SB), β‐Cyclodextrin‐Suxibuzone conjugate (SB‐CD), and 50 nM Bortezomib were loaded onto a precast 4%–12% polyacrylamide gel (Bolt, Invitrogen, Thermofisher) and electro‐transferred onto a nitrocellulose membrane in a wet transfer cell. Nonspecific binding was prevented by incubation with blocking buffer (LiCor, Biosciences). Membranes were incubated overnight at 4 °C with the following primary antibodies: Anti‐Ubiquitin antibody [EPR8830] (1:1000 dilution) and mouse monoclonal anti‐Actin 1:3000 dilution (SIGMA‐Aldrich). Secondary goat anti‐mouse labeled with IR dye 680 or IR dye 800 (1:20.000 Li‐COR 280 Biosciences, Lincoln, Nebraska, US) were used at RT for 45 min. Hybridization signals were detected with the Odyssey CLx Infrared Imaging System (LI‐COR Biosciences Lincoln, Nebraska, US).

##### Statistical Analysis

All data are expressed as mean ± SEM of three experiments performed at least in triplicate. Analyses were performed using GraphPad Prism (GraphPad Software, San Diego, CA, USA) statistical software. The one‐way ANOVA test, followed by Tukey's test, was applied. The value of *p* < 0.05 was considered statistically significant.

## Conflict of Interest

The authors declare no conflict of interest.

## Author Contributions

**Noemi Bognanni**: writing—review and editing, writing—original draft, visualization, validation, methodology, investigation, data curation. **Stefania Zimbone**: writing—review and editing, methodology, investigation, data curation. **Maria Laura Giuffrida**: methodology, investigation, data curation. **Giuseppe Di Natale**: writing—review and editing, methodology, investigation, data curation. **Danilo Milardi**: supervision, writing—review and editing, funding acquisition, resources. **Graziella Vecchio**: supervision, writing—review and editing, funding acquisition, resources, conceptualization. **Valeria Lanza**: writing—review and editing, writing—original draft, methodology, investigation, data curation, conceptualization.

## Supporting information

Supplementary Material

## Data Availability

The data that support the findings of this study are available from the corresponding author upon reasonable request.
