# Supplementary material for: Unveiling the Potential of a New β‐Cyclodextrin‐Suxibuzone Conjugate in Proteasome Regulation
Source: ChemMedChem. 2025 Oct 16;20(23):e202500401. doi: 10.1002/cmdc.202500401 (PMC12677842; doi:10.1002/cmdc.202500401)
Supplement: Supplementary file 1 — Supplementary Material [file CMDC-20-e202500401-s001.pdf]

Supplementary Information

Unveiling the Potential of a New β-Cyclodextrin-Suxibuzone Conjugate in Proteasome Regulation

Noemi Bognanni[b], Stefania Zimbone[a], Marialaura Giuffrida[a], Giuseppe Di Natale[a], Danilo Milardi[a], Graziella Vecchio[b] Valeria Lanza[a],

[a]Istituto di Cristallografia - CNR Sede Secondaria di Catania, Via P. Gaifami 18, 95126 Catania, Italy; [b]Dipartimento di Scienze Chimiche, University of Catania, V.le A.Doria, 6, 95125. Catania Italy

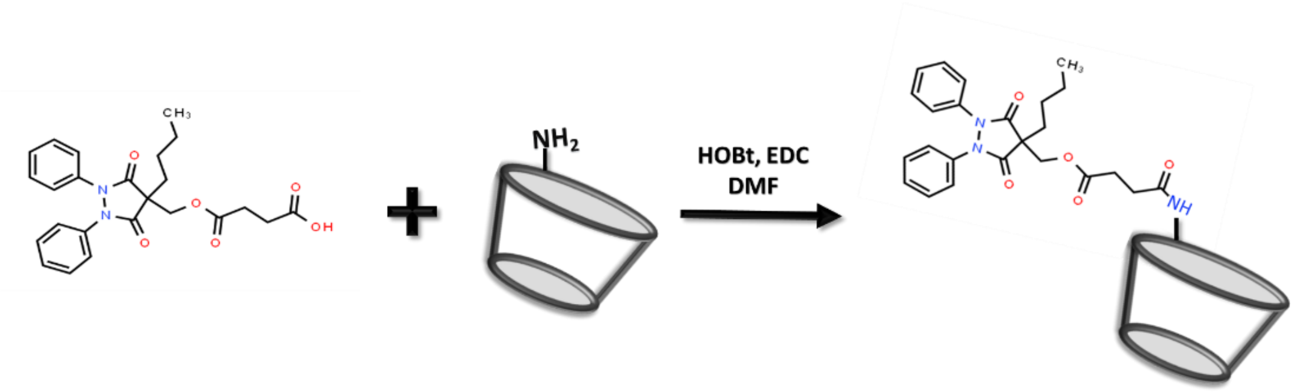

Figure S1. Synthetic scheme of SB-CD conjugate

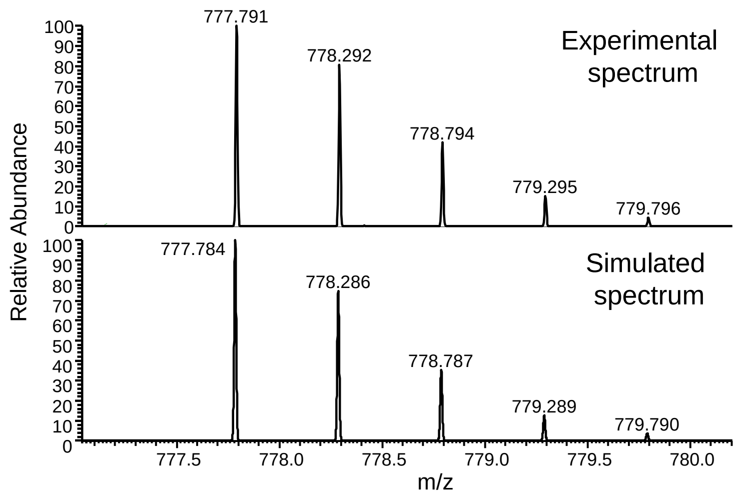

Figure S2. Mass spectra showing the comparison between the experimental and simulated isotopic distribution of the m/z peak corresponding to the SB-CD. Simulated spectrum was calculated for SB-CD molecular formula: C66H95O39N3.

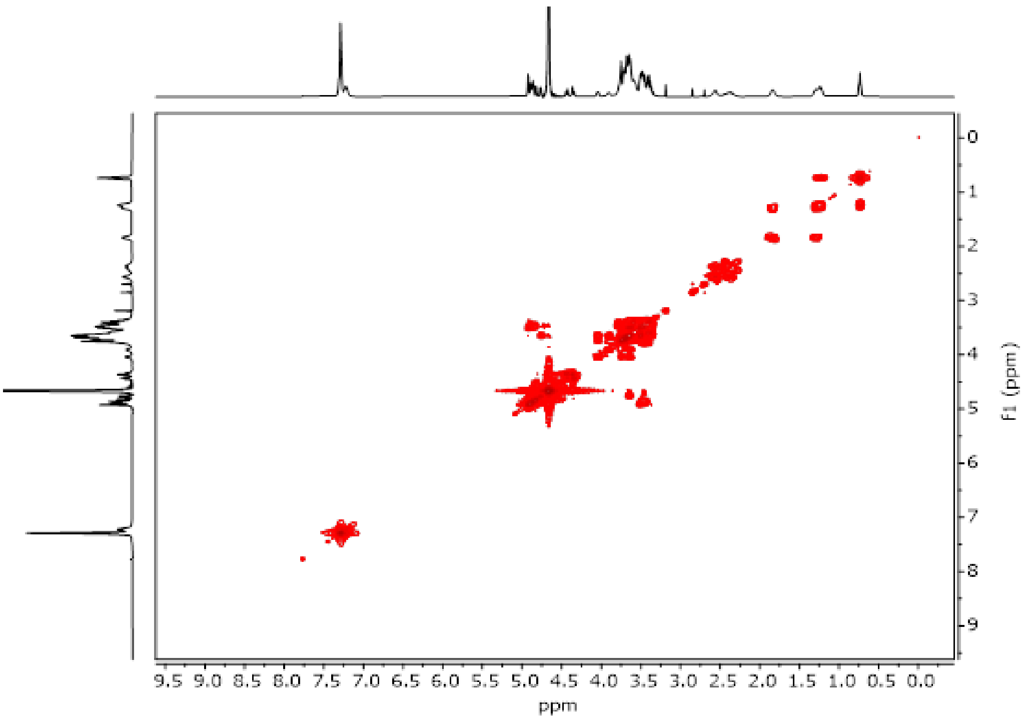

Figure S3. ***COSY spectrum of SB-CD in D$_2$O***

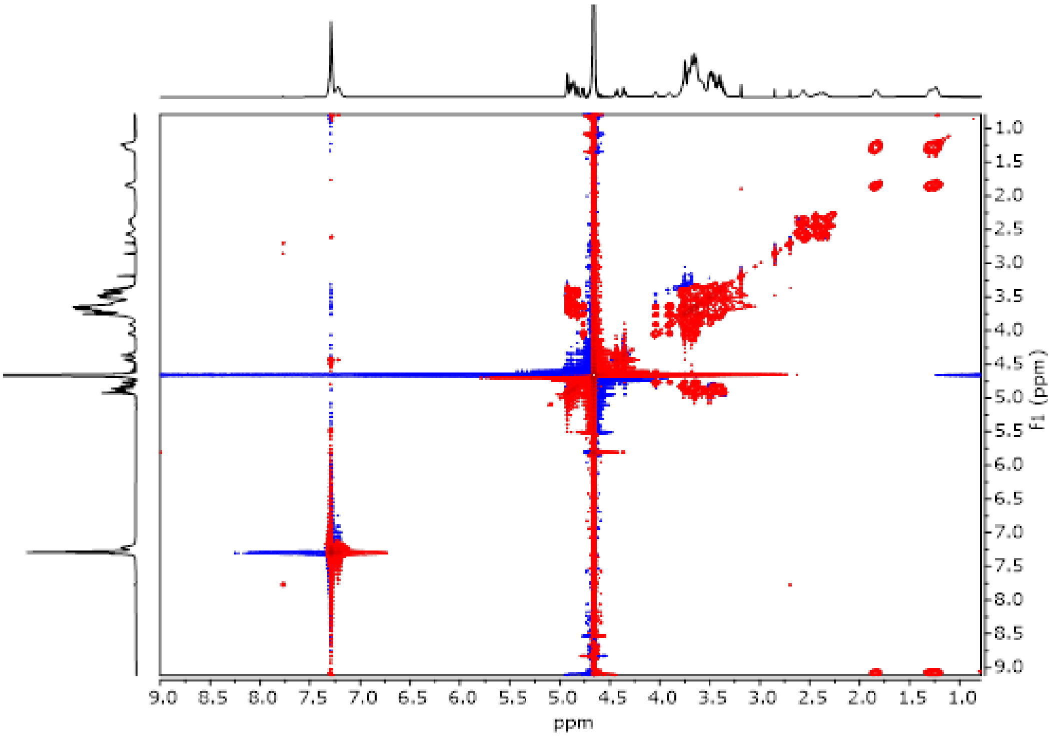

Figure S4. **TOCSY** ***spectrum of SB-CD in D$_2$O***

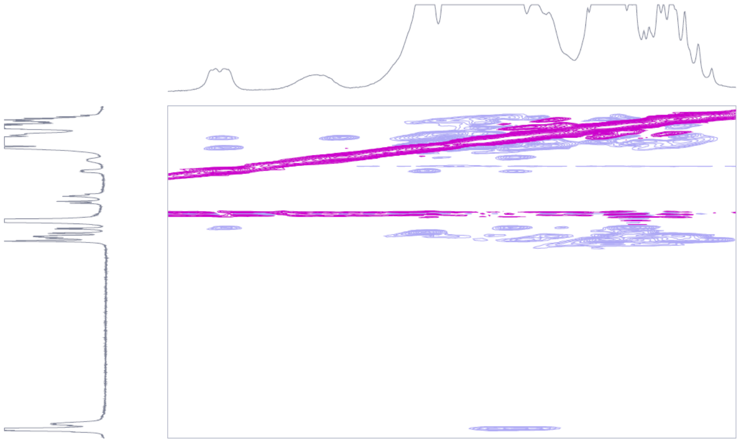

Figure S5. ROESY ***spectrum of SB-CD in*** D_2O

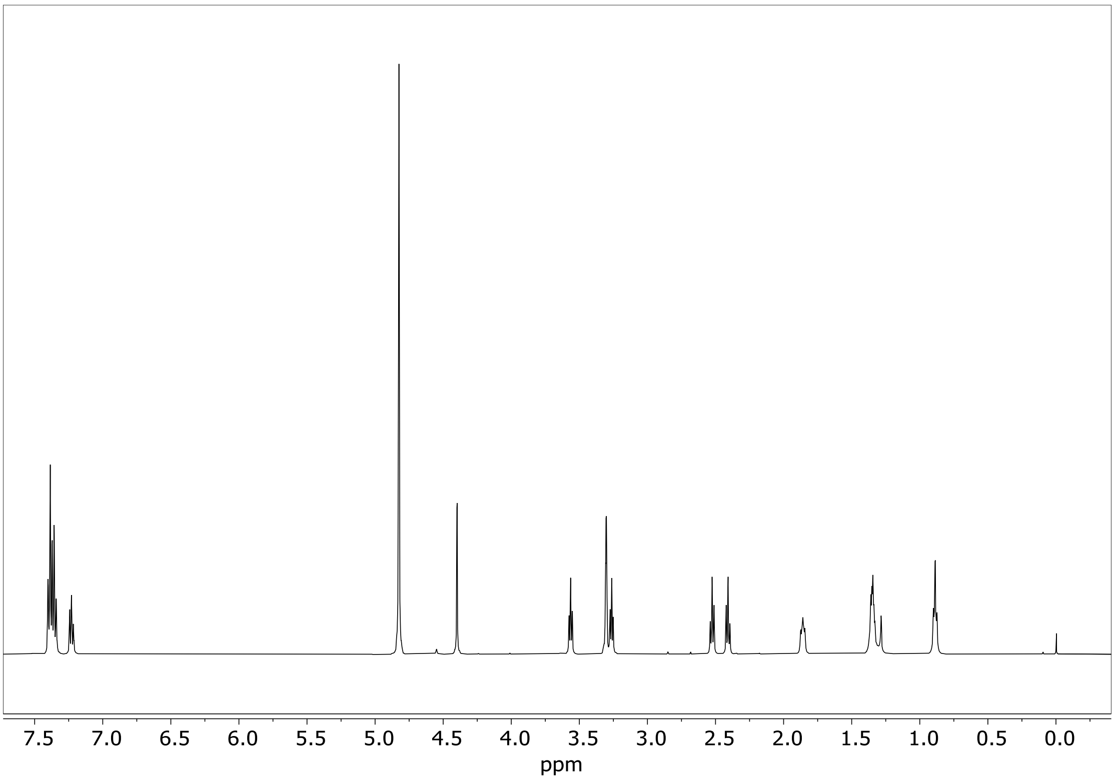

Figure S6. ^{1}**H NMR** ***spectrum of SB-ETA in*** CD_3OD

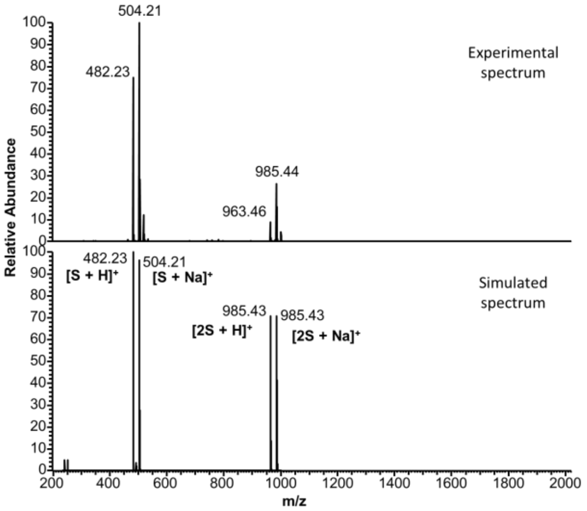

Figure S7: Mass spectra showing the comparison between the experimental and simulated isotopic distribution of the m/z peak corresponding to the SB-ETA.

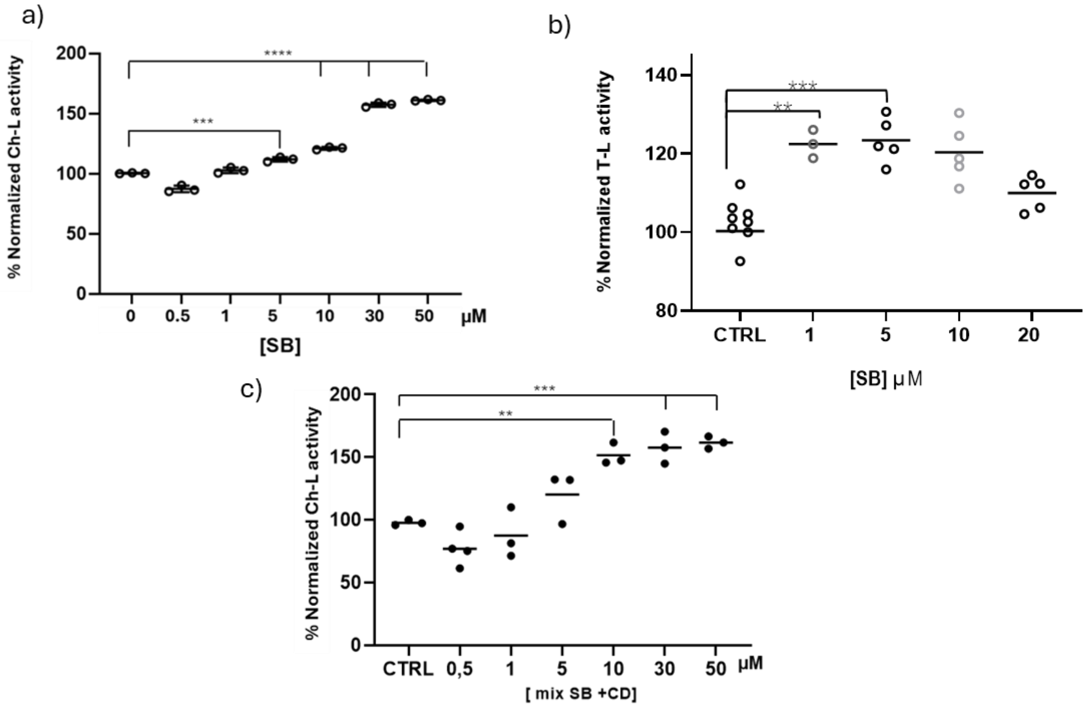

Figure S8: a) Ch-L and T-L activities of the h20S proteasome by different solutions of: a) and b) SB, in the concentration range 0-50 μM; c) Ch-L activity of 20S proteasome in presence of mixture SB and CD (1:1). Graphs represent the mean±SEM of three independent experiments with n=3. ****P<0.001, ***P<0.01, **P<0.05 vs control.

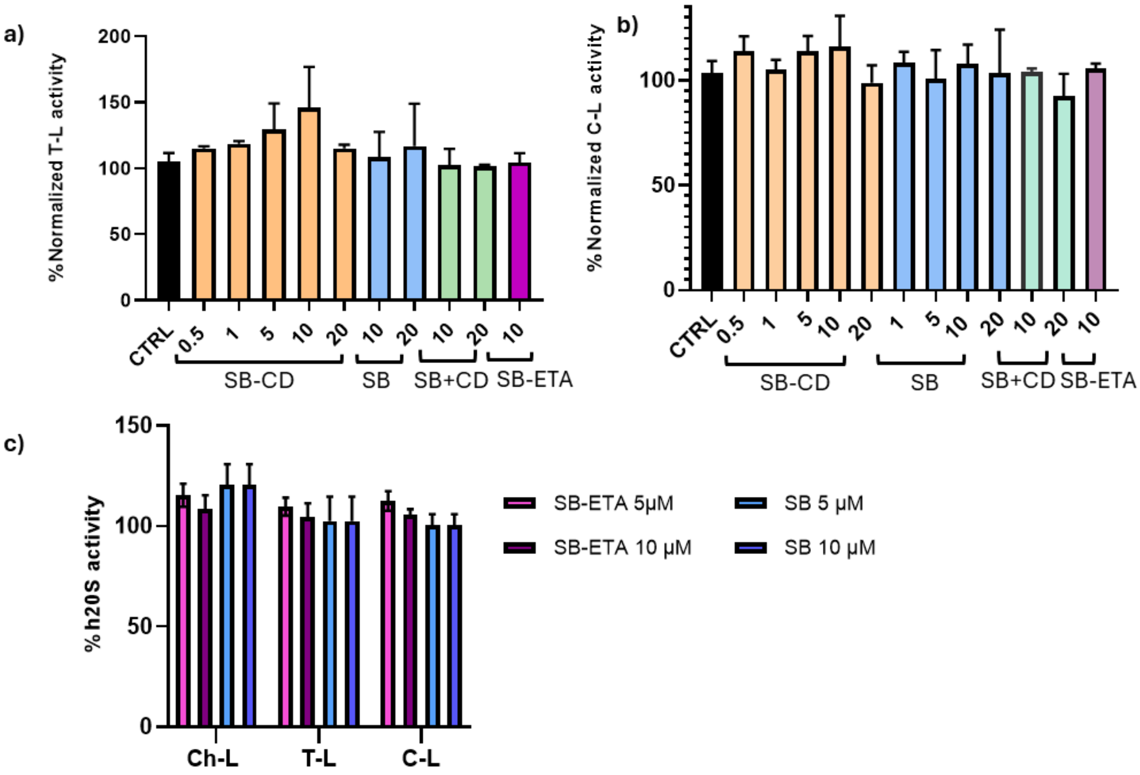

Figure S9: a) T-L activity and b) C-L activity of the h-20S proteasome in the presence of different solutions of SB-CD, SB, SB+CD, and SB-ETA; c) comparison of Ch-L, T-L, and C-L activities of SB and SB-ETA at concentrations of 5 and 10 μM. The bars indicate the mean ± standard deviation of three experiments (n=3)

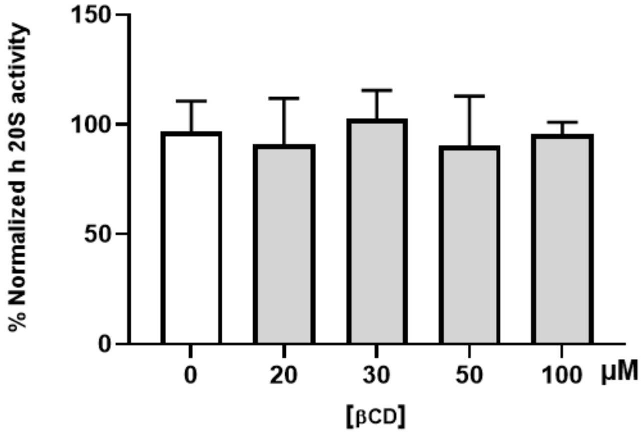

Figure S10: a) Normalized ChT-L activity of the h20S proteasome vs control in presence of β-cyclodextrin (range 0-100 μM).

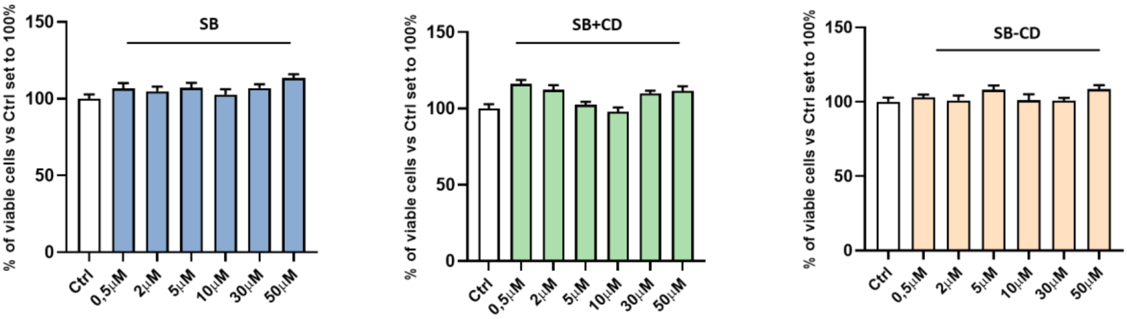

Figure S11: MTT assay of fully differentiated SH-SY5Y treated for 48 hours with increasing concentration of SB, SB+CD and SB-CD (0.5, 2, 5, 10, 30, 50 μM). Bars represent means ±SEM of three independent experiments with n = 3 each.
